# Serum AZD7442 (tixagevimab–cilgavimab) concentrations and *in vitro* IC_50_ values predict SARS‐CoV‐2 neutralising antibody titres

**DOI:** 10.1002/cti2.1517

**Published:** 2024-06-13

**Authors:** Lindsay E Clegg, Oleg Stepanov, Sam Matthews, Tom White, Seth Seegobin, Steven Thomas, Kevin M Tuffy, Mats Någård, Mark T Esser, Katie Streicher, Taylor S Cohen, Anastasia A Aksyuk

**Affiliations:** ^1^ Clinical Pharmacology and Quantitative Pharmacology, Clinical Pharmacology and Safety Sciences, R&D AstraZeneca Gaithersburg MD USA; ^2^ Clinical Pharmacology and Quantitative Pharmacology, Clinical Pharmacology and Safety Sciences, R&D AstraZeneca Cambridge UK; ^3^ Biometrics, Vaccines & Immune Therapies, BioPharmaceuticals R&D AstraZeneca Cambridge UK; ^4^ Biometrics, Vaccines & Immune Therapies, BioPharmaceuticals R&D AstraZeneca Durham NC USA; ^5^ Translational Medicine, Vaccines & Immune Therapies, BioPharmaceuticals R&D AstraZeneca Gaithersburg MD USA; ^6^ Vaccines & Immune Therapies, BioPharmaceuticals R&D AstraZeneca Gaithersburg MD USA

**Keywords:** authentic virus assay, AZD7442 (tixagevimab–cilgavimab), COVID‐19 monoclonal antibodies, pharmacokinetics, pseudovirus assay, SARS‐CoV‐2 neutralising antibody titres

## Abstract

**Objectives:**

The evolution of severe acute respiratory syndrome coronavirus 2 (SARS‐CoV‐2) necessitates rapid methods for assessing monoclonal antibody (mAb) potency against emerging variants. Authentic virus neutralisation assays are considered the gold standard for measuring virus‐neutralising antibody (nAb) titres in serum. However, authentic virus‐based assays pose inherent practical challenges for measuring nAb titres against emerging SARS‐CoV‐2 variants (e.g. storing infectious viruses and testing at biosafety level‐3 facilities). Here, we demonstrate the utility of pseudovirus neutralisation assay data in conjunction with serum mAb concentrations to robustly predict nAb titres in serum.

**Methods:**

SARS‐CoV‐2 nAb titres were determined via authentic‐ and lentiviral pseudovirus‐based neutralisation assays using serological data from three AZD7442 (tixagevimab–cilgavimab) studies: PROVENT (NCT04625725), TACKLE (NCT04723394) and a phase 1 dose‐ranging study (NCT04507256). AZD7442 serum concentrations were assessed using immunocapture. Serum‐based half‐maximal inhibitory concentration (IC_50_) values were derived from pseudovirus nAb titres and serum mAb concentrations, and compared with *in vitro* IC_50_ measurements.

**Results:**

nAb titres measured via authentic‐ and lentiviral pseudovirus‐based neutralisation assays were strongly correlated for the ancestral SARS‐CoV‐2 virus and SARS‐CoV‐2 Alpha. Serum AZD7442 concentrations and pseudovirus nAb titres were strongly correlated for multiple SARS‐CoV‐2 variants with all Spearman correlation coefficients ≥ 0.78. Serum‐based IC_50_ values were similar to *in vitro* IC_50_ values for AZD7442, for ancestral SARS‐CoV‐2 and Alpha, Delta, Omicron BA.2 and Omicron BA.4/5 variants.

**Conclusions:**

These data highlight that serum mAb concentrations and pseudovirus *in vitro* IC_50_ values can be used to rapidly predict nAb titres in serum for emerging and historical SARS‐CoV‐2 variants.

## Graphical abstract

In the analyses reported herein, we use data from clinical studies of the coronavirus disease 2019 (COVID‐19) monoclonal antibody (mAb) combination AZD7442 (tixagevimab–cilgavimab) to demonstrate the utility of pseudovirus neutralisation assay data in conjunction with mAb serum concentrations to predict neutralising antibody titres against emergent severe acute respiratory syndrome coronavirus 2 (SARS‐CoV‐2) variants. Our findings offer a strategy to streamline the clinical evaluation of mAbs targeting SARS‐CoV‐2 and have the potential to significantly decrease the time from discovery to clinical deployment of the next generation of COVID‐19 mAbs.

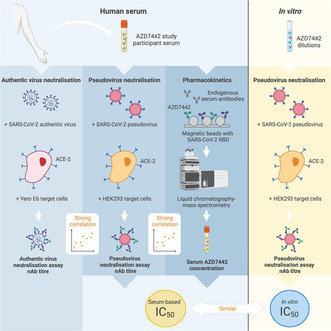

## Introduction

The coronavirus disease 2019 (COVID‐19) pandemic has featured waves of infection driven by the continued emergence of novel severe acute respiratory syndrome coronavirus 2 (SARS‐CoV‐2) variants, each time with enhanced humoral immune evasion.^1^, ^2^, ^3^, ^4^ Monoclonal antibodies (mAbs), predominantly selected and developed for their neutralising potency against ancestral SARS‐CoV‐2, have played an important role in the prevention and treatment of COVID‐19 in vulnerable populations, including immunocompromised individuals.^5^, ^6^

Serological studies have shown that anti‐SARS‐CoV‐2 neutralising antibody (nAb) titres are a key measure of immune protection following the administration of COVID‐19 vaccines or mAbs, as they correlate with protection against symptomatic and severe infection.^7^, ^8^, ^9^, ^10^ The neutralisation titres of COVID‐19 mAbs in serum are often measured using authentic virus‐based neutralisation assays (e.g. the plaque reduction neutralisation test [PRNT]), which are considered the gold standard for measuring antibody titres for many viral diseases.^11^ However, the use of authentic, virus‐based assays poses inherent practical challenges, including the need to source, isolate and maintain large stocks of viruses for all variants, and perform testing in biosafety level‐3 (BSL 3) facilities.^11^, ^12^, ^13^

**Figure 1:**
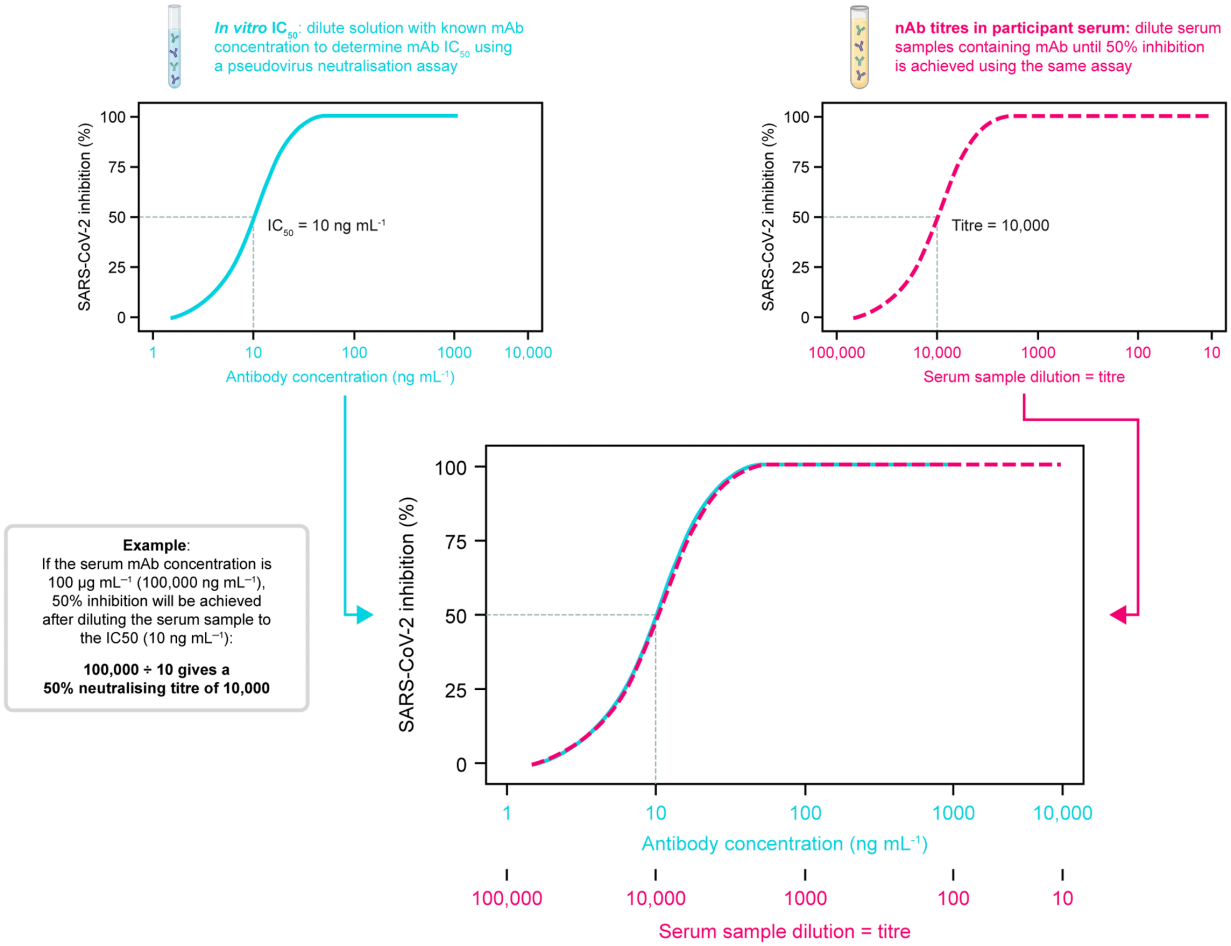
Calculation of predicted nAb titres. This schematic illustrates how *in vitro* IC_50_ values and serum mAb concentrations can be used to predict nAb titres if measured using the same pseudovirus neutralisation assay. IC_50_, half‐maximal inhibitory concentration; mAb, monoclonal antibody; nAb, neutralising antibody; SARS‐CoV‐2, severe acute respiratory coronavirus 2.

The rapid evolution of SARS‐CoV‐2 has necessitated an equally rapid method for assessing the potency of mAbs against newly circulating variants, to determine whether existing mAbs will provide protection against emerging variants, particularly in individuals at high risk of COVID‐19.^5^, ^14^ Several pseudovirus‐based assays have been used in registrational studies for COVID‐19 vaccines and mAbs.^15^, ^16^, ^17^, ^18^, ^19^ Such assays offer increased reproducibility and logistical advantages over authentic virus neutralisation assays.^11^, ^12^ For example, the lentiviral pseudovirus neutralisation assay utilises a non‐replicating, genetically modified lentiviral construct encoding the SARS‐CoV‐2 spike protein and a luciferase reporter gene.^12^, ^13^ Co‐incubation of these lentiviral pseudovirions with mAb‐containing serum or directly with mAbs (*in vitro*) and an infection‐permissive cell type (e.g. cells expressing the angiotensin‐converting enzyme 2 [ACE2] receptor) allows for a readily reproducible assessment of SARS‐CoV‐2 nAb potency, measured as a half‐maximal inhibitory concentration (IC_50_).^11^, ^12^, ^13^ Additionally, lentiviral pseudovirions can be consistently produced and rapidly adapted to new viral variants using public viral sequence data without the need to isolate infectious virus. Furthermore, COVID‐19 mAb serum concentrations, normalised by neutralisation potency IC_50_ values against different variants, can be used to estimate serum nAb titres, as described in Figure 1. These ‘predicted nAb titres’, calculated as serum mAb concentration divided by *in vitro* IC_50_, have been observed to correlate with protection against symptomatic SARS‐CoV‐2 infection.^20^ However, there remains a need to determine the degree of agreement between authentic virus and pseudovirus neutralisation assays, as well as to explore the reliability of using serum mAb concentration in combination with *in vitro* IC_50_ values to predict nAb titres against emerging variants.

AZD7442 (tixagevimab–cilgavimab) is composed of two extended half‐life (M252Y/S254T/T256E [YTE]‐modified) mAbs that bind to distinct non‐overlapping epitopes on the SARS‐CoV‐2 spike receptor‐binding domain, allowing for direct virus neutralisation.^21^, ^22^ The AZD7442 mAb combination received emergency use authorisation (EUA) for COVID‐19 pre‐exposure prophylaxis in the United States in December 2021 and subsequently received authorisations for pre‐exposure prophylaxis or treatment in the EU, Canada, Japan, Australia and other markets; however, with the emergence of resistant Omicron variants, the US EUA for AZD7442 was suspended in 2023.^23^ Despite this, throughout its development, AZD7442 safety, efficacy, pharmacokinetics (PK) and nAb titres to the ancestral SARS‐CoV‐2 virus have been characterised in clinical trials.^17^, ^24^, ^25^ Such findings include demonstration of AZD7442 efficacy in two phase 3 studies, in the prevention of symptomatic COVID‐19 (PROVENT; NCT04625725)^17^ and in the prevention of hospitalisation among outpatients with COVID‐19 (TACKLE; NCT04723394).^25^ Additionally, AZD7442 has been shown to possess a range of *in vitro* IC_50_ values against ancestral, Alpha, Delta and Omicron SARS‐CoV‐2 variants.^23^, ^26^ Therefore, AZD7442 serum levels and nAb titres against different variants provide an ideal dataset to evaluate correlation between different neutralisation assay methods and correlation between mAb serum concentrations and variant‐specific nAb titres, as well as for determining the utility of serum concentrations and *in vitro* IC_50_ measurements to predict nAb titres.

Here, we first present an analysis of correlations between SARS‐CoV‐2 nAb titres measured by authentic‐ and pseudovirus‐based neutralising assays using serological data from PROVENT,^17^ TACKLE^25^ and the phase 1 first‐in‐human study of AZD7442.^24^ Second, we use data from PROVENT to evaluate the relationship between serum AZD7442 concentrations and pseudovirus‐derived nAb titres against multiple SARS‐CoV‐2 variants. Third, we evaluate whether serum‐derived IC_50_ values are similar to *in vitro* IC_50_ measurements. Finally, we use serum concentrations and *in vitro* IC_50_ measurements to predict nAb titres and compare these to measured nAb titres across five SARS‐CoV‐2 variants.

## Results

### Correlation between authentic‐ and pseudovirus‐based SARS‐CoV‐2 neutralisation assays

**Figure 2:**
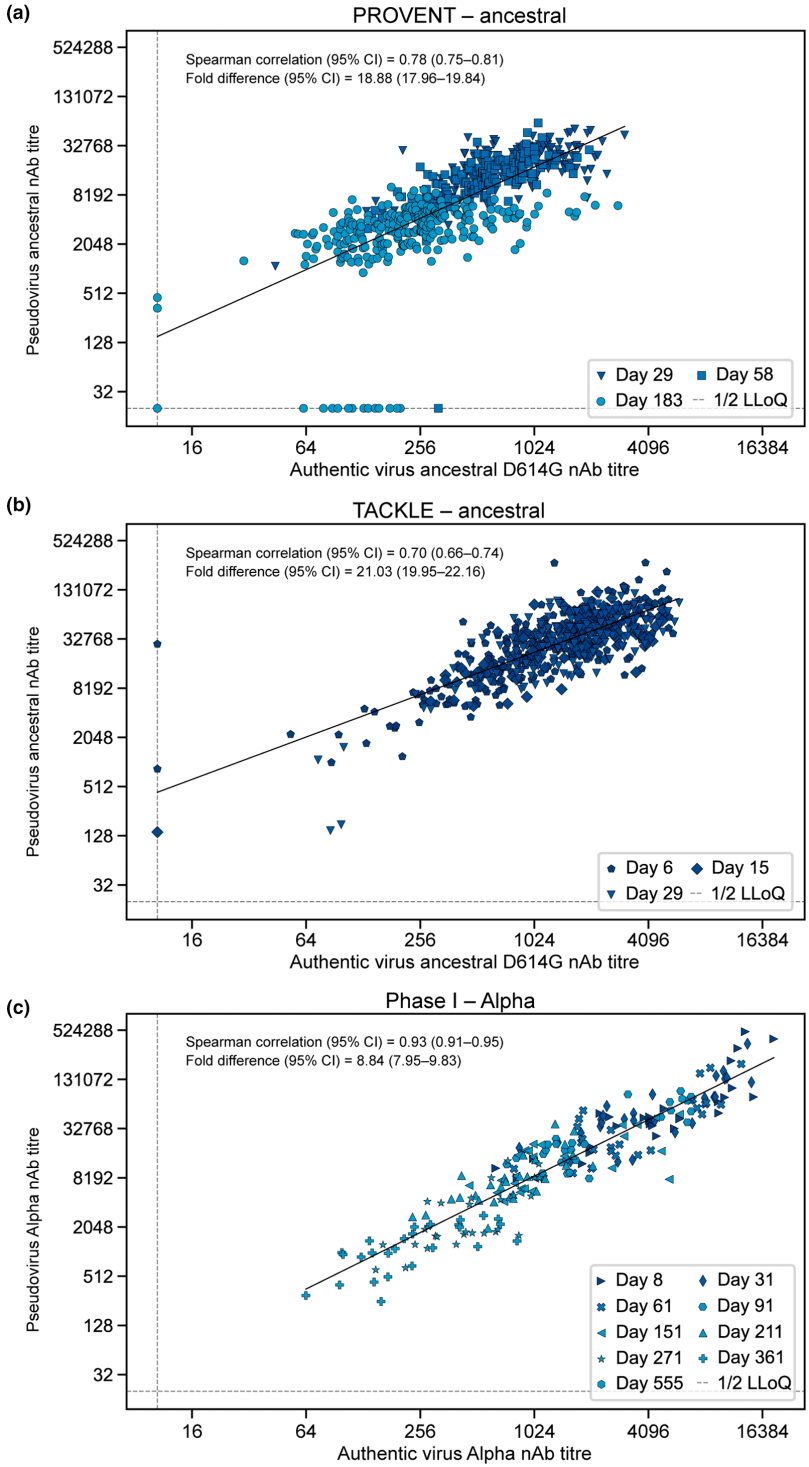
Correlation of SARS‐CoV‐2 nAb titres measured by authentic virus versus pseudovirus neutralisation assays. *Post hoc* correlation analyses depicting the relationship between **(a, b)** ancestral SARS‐CoV‐2 and **(c)** SARS‐CoV‐2 Alpha nAb titres determined by pseudovirus (*y*‐axis) and authentic virus (*x*‐axis) assays in serum samples collected from the **(a)** PROVENT (ADZ7442 300 mg IM), **(b)** TACKLE (600 mg IM) and **(c)** phase 1 first‐in‐human (300 mg, 1000 mg or 3000 mg IV) clinical studies. Data points indicate matched authentic‐ and pseudovirus‐derived nAb titres (observations) from the same study participants. Participant sample availability in **(a)** PROVENT was Day 29 (*n =* 168), Day 58 (*n =* 163) and Day 183 (*n =* 248); **(b)** TACKLE was Day 6 (*n =* 284), Day 15 (*n =* 107) and Day 29 (*n =* 178); **(c)** the phase 1 study was Day 8 (*n =* 28), Day 31 (*n =* 28), Day 61 (*n =* 28), Day 91 (*n =* 27), Day 151 (*n =* 27), Day 211 (*n =* 28), Day 271 (*n =* 29), Day 361 (*n =* 26) and EDV (*n =* 1). CI, confidence interval; EDV, early discontinuation visit; IM, intramuscular; IV, intravenous; LLoQ, lower limit of quantification; nAb, neutralising antibody; SARS‐CoV‐2, severe acute respiratory coronavirus 2.

To better understand whether the pseudovirus‐based SARS‐CoV‐2 neutralisation assays that were used to support the AZD7442 clinical development programme correlated with authentic virus‐based neutralisation assays, we evaluated the correlation between nAb titres generated in the two assays, when measured in the same participants. The study designs for the PROVENT, TACKLE and phase 1 first‐in‐human studies are detailed in the Methods. Correlations between nAb titre levels (PRNT_80_ for authentic virus assay and 50% infectious dose [ID_50_] for pseudovirus assay; see the Methods) derived from authentic‐ and pseudovirus‐based neutralising assays were evaluated using serum samples obtained from study participants (Figure 2; Table 1).^27^

**Table 1:** Correlations between authentic versus pseudovirus‐based nAb titres in serum samples from AZD7442 clinical studies

AZD7442 trial	Authentic SARS‐CoV‐2 virus	*n* ^a^	Spearman correlation^b^ (95% CI)	Repeated measures correlation^c^ (95% CI)
PROVENT (phase III)	Ancestral D614G	579	0.78 (0.75–0.81)	0.77 (0.74–0.81)
TACKLE (phase III)	Ancestral D614G	569	0.70 (0.66–0.74)	0.78 (0.75–0.82)
First‐in‐human (phase I)	Alpha	222	0.93 (0.91–0.95)	0.93 (0.91–0.95)

CI, confidence interval; LLoQ, lower limit of quantification; nAb, neutralising antibody; SARS‐CoV‐2, severe acute respiratory coronavirus 2.

^a^ Number of available post‐baseline observations above LLoQ for both authentic virus and pseudovirus nAb titres.

^b^ As log_10_‐transformed nAbs are skewed, the bias‐adjusted Spearman correlation was used following Fisher's Z transformation for the 95% CI.

^c^ The correlation coefficient estimate for the repeated measurement data is calculated following the methods by Hamlett *et al*.,^46^ and the 95% CI for the correlation coefficient was estimated using the normal approximation method by Shen and Lu.^47^

A strong correlation (defined as Spearman correlation coefficient ≥ 0.7) was observed between nAb titres against ancestral SARS‐CoV‐2 D614G authentic virus and ancestral SARS‐CoV‐2 pseudovirus in PROVENT study participants (Spearman correlation coefficient: 0.78 [95% confidence interval [CI] 0.75–0.81]) (Figure 2a). The dataset included matched samples obtained throughout the course of the study (Days 29, 58 and 183). An analysis in TACKLE study participants (Days 6, 15 and 29) also revealed a strong correlation between ancestral SARS‐CoV‐2 D614G authentic and ancestral SARS‐CoV‐2 pseudovirus nAb titres (Spearman correlation coefficient: 0.70 [95% CI 0.66–0.74]) (Figure 2b). An important distinction between the PROVENT and TACKLE studies was the timing of AZD7442 administration; the PROVENT study evaluated AZD7442 300 mg via intramuscular (IM) injection for pre‐exposure prophylaxis, whereas the TACKLE study examined AZD7442 600 mg IM as an outpatient treatment after a confirmed SARS‐CoV‐2 infection. In this context, our data from TACKLE participants illustrate that a strong correlation between authentic and pseudovirus nAb titres is maintained over time in the outpatient treatment setting, despite the likely presence of nAbs from participant immune responses following SARS‐CoV‐2 infection and from AZD7442.

We also evaluated authentic and pseudovirus nAb titres for SARS‐CoV‐2 Alpha to expand the correlation analysis to another SARS‐CoV‐2 variant. Analysis of samples from the AZD7442 phase 1 first‐in‐human study participants showed a strong correlation between authentic and pseudovirus nAb titres (Spearman correlation coefficient: 0.93 [95% CI 0.91–0.95]) (Figure 2c). These samples came from healthy participants who received AZD7442 300 mg IM, 300 mg intravenous (IV), 1000 mg IV or 3000 mg IV, and therefore, the range of serum AZD7442 concentrations was broader than in the phase 3 studies. The dataset included matched samples obtained throughout the course of the study (Days 8, 31, 61, 91, 151, 211, 271 and 361). The correlation remained consistent across the range of serum AZD7442 concentrations and collection times following dosing.

As expected, we observed a fold difference between authentic and pseudovirus assays (ranging from 9‐fold to 20‐fold across the three groups), which is likely a result of differences between assay outputs (PRNT_80_ and ID_50_) and setup (e.g. viral replication and growth kinetics). However, Spearman correlation coefficients were ≥ 0.7 in each of these analyses despite the different clinical settings (i.e. pre‐exposure prophylaxis and post‐infection treatment), and across various doses of AZD7442 (Table 1). Taken together, these data provide strong evidence that authentic‐ and pseudovirus‐derived nAb titres correlate and are equally suitable for assessing nAb titres across study populations.

### Correlations between serum AZD7442 concentrations and pseudovirus‐derived nAb titres across SARS‐CoV‐2 variants in PROVENT study participants

**Figure 3:**
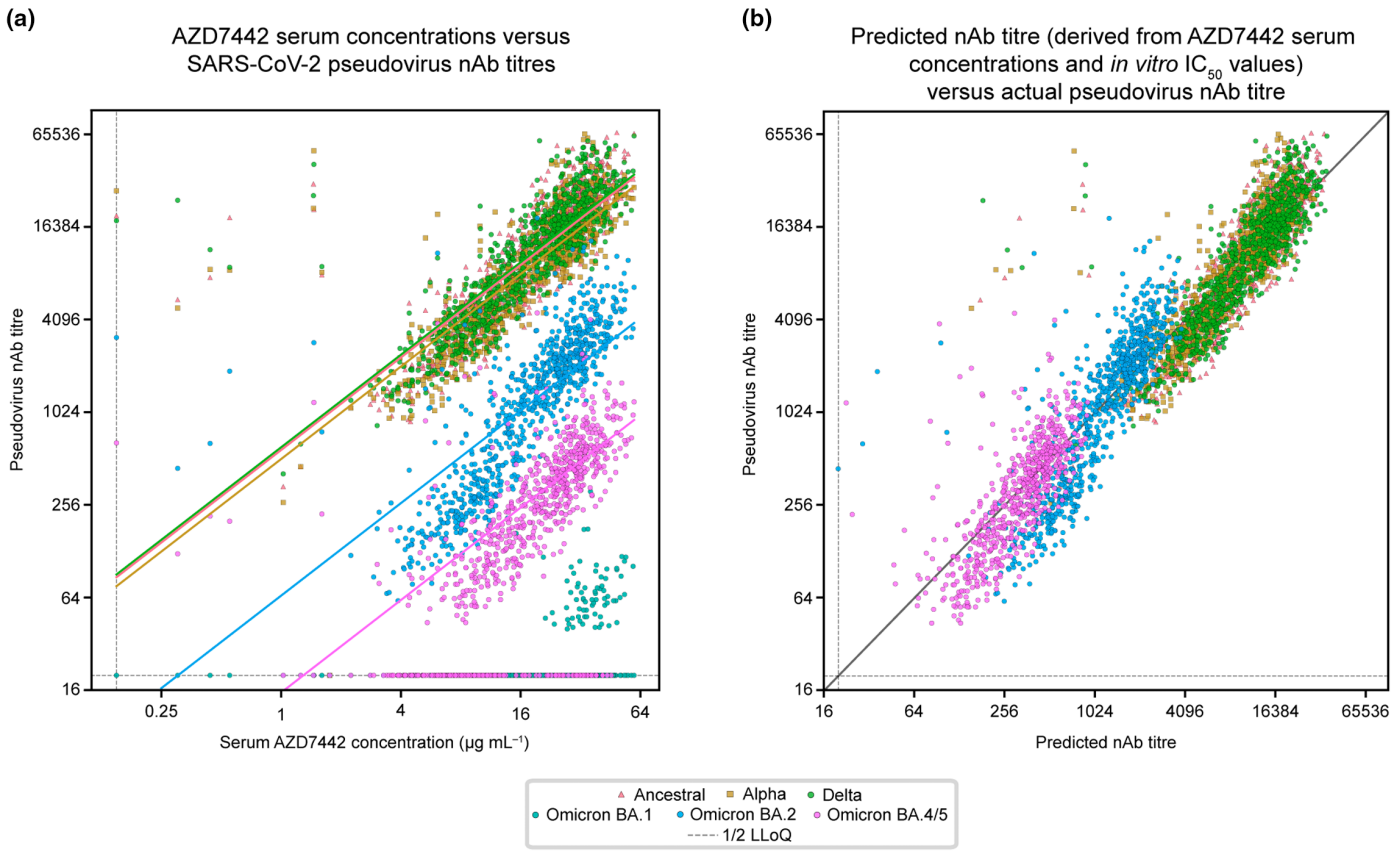
Correlation between AZD7442 serum concentrations and SARS‐CoV‐2 pseudovirus nAb titres in PROVENT. *Post hoc* correlation analysis depicting the relationship between nAb titres determined by **(a)** pseudovirus assay and serum concentrations of AZD7442; or **(b)** predicted nAb titres. Data points depict matched PK and nAb sample pairs (observations) from the same PROVENT study participants. Colours indicate different SARS‐CoV‐2 variants. PK and nAb titre measurements below the LLoQ were imputed to half of the LLoQ for visualisation in **(a)**. LLoQ, lower limit of quantification; nAb, neutralising antibody; PK, pharmacokinetic.

To explore the relationship between serum AZD7442 concentration and nAb titres, we used a subset of matched samples from approximately 300 PROVENT study participants with pseudovirus nAb titres against ancestral SARS‐CoV‐2, as well as five later variants (Figure 3). A linear relationship was observed between serum AZD7442 concentrations and pseudovirus nAb titres, with Spearman correlation coefficients ≥ 0.78, illustrating a strong correlation between serum AZD7442 concentrations and reported nAb titres across multiple SARS‐CoV‐2 variants (Table 2). Serum AZD7442 concentrations and pseudovirus nAb titres displayed similar correlations and patterns of variability over time for ancestral SARS‐CoV‐2, as for the Alpha, Delta, Omicron BA.2 and Omicron BA.4/5 variants (Supplementary figure 1). Serum AZD7442 concentration‐nAb titre pairs with a measurement below the lower limit of quantification (LLoQ) for the PK assay and nAb assay were excluded from this analysis, as measurements without a quantifiable result cannot inform the numerical relationship between these two metrics (e.g. serum‐based IC_50_). However, a strong rank‐based correlation (Spearman correlation coefficients > 0.70) was maintained even when samples below the LLoQ were imputed to 1/2 the LLoQ (Supplementary table 1). For Omicron BA.1, as most observations were below the LLoQ in the pseudovirus nAb assay, it was deemed there was not sufficient data to evaluate the correlation for this variant. As expected, because of decreasing levels of AZD7442 over time, the proportion of samples with nAb titres below the LLoQ of the neutralisation assay increased at Day 183 for both Omicron BA.2 and BA.4/5, when nAb titres were generally lower than those for the ancestral, Alpha and Delta variants, which is consistent with the established potency of AZD7442 against these variants.^23^, ^26^

**Table 2:** Spearman correlations for serum AZD7442 concentrations and pseudovirus nAb titres across SARS‐CoV‐2 variants in PROVENT study participants

SARS‐CoV‐2 virus	*n* ^a^	Spearman correlation (95% CI)^b^
Ancestral	854	0.89 (0.88–0.91)
Alpha	855	0.87 (0.85–0.89)
Delta	860	0.87 (0.86–0.89)
Omicron BA.1^c^	74	NE
Omicron BA.2	809	0.87 (0.86–0.89)
Omicron BA.4/5	670	0.78 (0.75–0.81)

CI, confidence interval; LLoQ, lower limit of quantification; nAb, neutralising antibody; NE, not evaluable; SARS‐CoV‐2, severe acute respiratory coronavirus 2.

^a^ Number of available post‐baseline observations above the LLoQ for both pseudovirus nAb titres and serum concentration for calculation of Spearman correlations.

^b^ As log_10_‐transformed nAbs and serum concentrations are skewed, the bias‐adjusted Spearman correlation was used following Fisher's Z transformation for the 95% CI.

^c^ As a high percentage of observations were below the LLoQ for Omicron BA.1, it was deemed there were not sufficient data to evaluate the serum concentration and nAb correlation.

### Comparison between serum‐based and *in vitro* AZD7442 IC_50_ values

Although use of *in vitro* IC_50_ values to evaluate the potency of mAbs against SARS‐CoV‐2 variants is widely accepted, there are limited data describing how well *in vitro* IC_50_ values correlate with nAb titres for different SARS‐CoV‐2 variants. Therefore, we compared *in vitro* IC_50_ values obtained from pseudovirus incubated with purified mAb, with the IC_50_ values derived from the sera of clinical trial participants. As serum pseudovirus nAb titres reflect the sample dilution that results in 50% inhibition of reporter signal, we could estimate serum‐based AZD7442 IC_50_ values for each variant based on the relationship between serum AZD7442 concentrations and pseudovirus nAb titres measured in matched serum samples (i.e. the serum‐based IC_50_ value is the serum AZD7442 concentration predicted to result in 50% neutralisation in the pseudovirus nAb assay, based on clinical samples [see Figure 1 and the Methods for further details]).

We compared these serum‐based AZD7442 IC_50_ values against each SARS‐CoV‐2 virus (ancestral and five variants) with *in vitro* AZD7442 IC_50_ values obtained from purified mAb in the same neutralisation assay. For the four viruses with sufficient available data (conclusions could not be made for Omicron BA.1), serum‐based AZD7442 IC_50_ values were similar to the corresponding *in vitro* IC_50_ values across a wide range of IC_50_ values (~2–70 ng mL^─1^) (Table 3). To evaluate whether the relationship between serum AZD7442 concentrations and pseudovirus nAb titres was maintained in participants with known SARS‐CoV‐2 infection, the Spearman correlation coefficient and serum‐based IC_50_ for the ancestral SARS‐CoV‐2 virus were evaluated in 426 participants with available data from the TACKLE study. As shown in Supplementary figure 2, a strong correlation (Spearman correlation coefficient 0.92) and consistent serum‐based IC_50_ (1.85 ng mL^─1^) was also observed in this dataset. Our analysis of *in vitro* IC_50_ values, serum AZD7442 concentrations and pseudovirus nAb titres suggests that: (1) there is a strong correlation between serum AZD7742 concentrations and neutralising titres in participant serum samples; (2) this correlation is observed across multiple SARS‐CoV‐2 variants; and (3) serum‐based AZD7442 IC_50_ values are generally similar to *in vitro* IC_50_ values.

**Table 3:** Comparison between serum‐based and *in vitro* AZD7442 IC_50_ values for ancestral SARS‐CoV‐2 and SARS‐CoV‐2 variants

SARS‐CoV‐2 virus	*n* ^a^	Serum‐based AZD7442 IC_50_ value, ng mL^−1^ (95% CI)^b^	*In vitro* AZD7442 IC_50_ value, ng mL^−1^ (STD)^c^
Ancestral	854	1.73 (1.66–1.81)	2.2 (0.7)
Alpha	855	1.98 (1.90–2.07)	2.1 (0.5)
Delta	860	1.66 (1.59–1.74)	2.2 (0.7)
Omicron BA.1^d^	74	NE	171.1 (59.9)
Omicron BA.2	809	15.25 (14.58–15.95)	9.8 (2.5)
Omicron BA.4/5	670	64.94 (61.91–68.12)	69.4 (24.2)

CI, confidence interval; IC_50_, half‐maximal inhibitory concentration; LLoQ, lower limit of quantification; nAb, neutralising antibody; NE, not evaluable; PK, pharmacokinetics; SARS‐CoV‐2, severe acute respiratory coronavirus 2; STD, standard deviation.

^a^ Number of available post‐baseline observations above the LLoQ for both pseudovirus nAb titres and serum concentration for calculation of serum‐based AZD7442 IC_50_ values.

^b^ Serum‐based AZD7442 IC_50_ values were calculated as detailed in Statistical analyses: Derivation of serum‐based IC_50_ values.

^c^ STD was based on estimated assay precision from two technical replicates.

^d^ As a high percentage of observations were below the LLoQ for Omicron BA.1, it was deemed there was not sufficient data to evaluate the IC_50_ and correlation.

### Predicted nAb titres

Lastly, to determine whether we could use AZD7442 serum concentrations and *in vitro* IC_50_ values to predict nAb titres against multiple variants, we compared ‘predicted nAb titres’, calculated as serum mAb concentration divided by *in vitro* IC_50_, to observed nAb titres against the ancestral virus and the Alpha, Delta and Omicron BA.1, BA.2 and BA.4/5 variants. The results show a consistent relationship between predicted and observed nAb titres across SARS‐CoV‐2 variants (Figure 3b), suggesting that serum mAb concentrations and *in vitro* neutralisation IC_50_ measurements can effectively estimate, or predict, post‐administration nAb titres. Notably, a small number of data points fell to the left of the diagonal line in Figure 3b, suggesting that these participants may have had an asymptomatic SARS‐CoV‐2 infection or received a COVID‐19 vaccine, resulting in higher than expected nAb titres.

## Discussion

Neutralising mAbs have played a significant role in both the prevention of, and treatment against, COVID‐19.^17^, ^25^ Although at the time of manuscript preparation AZD7442 is no longer authorised for COVID‐19 pre‐exposure prophylaxis in the United States, the neutralising activity of AZD7442 has been extensively examined throughout its history of use.^23^, ^26^ The use of antibody half‐life extension technologies such as the YTE and LS modifications have made long‐acting antibodies (LAABs) attractive options for pre‐exposure prophylaxis in populations who are unable to mount a sufficient immune response, such as those with immunocompromising conditions, transplant recipients and those taking powerful immunosuppressants.^17^, ^25^, ^28^, ^29^ As both prophylactic and treatment approaches for COVID‐19 rely inherently on the susceptibility of the SARS‐CoV‐2 virus to mAb neutralisation, it is important to have the ability to rapidly assess the neutralising potency of COVID‐19 mAbs against emerging viral variants.

The data presented herein support the use of pseudovirus‐derived nAb titres as a surrogate for authentic‐derived titres as a measurement of viral neutralisation in a clinical setting. This alternative approach presents an important opportunity for rapid development and assessment of mAbs targeting SARS‐CoV‐2, compared with the use of authentic virus‐based neutralisation assays, which present practical challenges in their development, validation and implementation against rapidly emerging SARS‐CoV‐2 variants. While potential differences in viral growth kinetics^30^ pose a challenge for any cross‐variant comparisons of authentic virus assays, lentiviral SARS‐CoV‐2 pseudoviruses are identical, with the exception of the spike sequence, across different variants; they are therefore more likely to have consistent assay parameters across variants. It is, however, important to note that the same type of assay and the same laboratory and/or vendor should be used for cross‐variant comparisons, because of differences in the numerical titre measurements with different assays.

In this report, we also demonstrate the suitability of predicted nAb titres as a surrogate for the assessment of serum neutralisation through a combination of robust correlation between serum AZD7442 concentrations and pseudovirus nAb titres from matched samples across five SARS‐CoV‐2 variants, and consistency of the associated serum‐based IC_50_ with *in vitro* AZD7442 IC_50_ values across a wide range of potencies (pseudovirus IC_50_ values of ~2–70 ng mL^─1^). Unlike nAb titres, mAb serum concentrations do not depend on a specific SARS‐CoV‐2 variant, are not influenced by prior vaccination or infection, and can be measured faster and with higher precision because of lower assay variability than nAb titres. Additionally, no clinical samples are required for pseudovirus *in vitro* neutralisation assays, allowing for high‐throughput assessments of mAbs against newly emergent variants as soon as the genetic sequences of the desired antigens are available.^11^, ^12^, ^13^ Therefore, estimating nAb titres based on mAb serum concentration in conjunction with *in vitro* IC_50_ values provides both a fast and ‘clean’ estimate of nAb levels related specifically to drug administration. This is key in facilitating rapid immunobridging studies by enabling enrolment of participants with varying levels of nAbs at baseline (i.e. both healthy and immunocompromised participants), allowing for faster recruitment and smaller required sample sizes.

Similar findings have been shown in the human immunodeficiency virus (HIV) field; there is a precedent for the use of serum drug concentrations normalised by IC_50_ to estimate nAb titres (referred to as the ‘inhibitory quotient’) for therapeutic drug monitoring.^31^, ^32^ Furthermore, an analysis of the broadly neutralising anti‐HIV envelope glycoprotein mAb, VRC01, illustrated that predicted nAb titres (calculated as VRC01 serum concentration/*in vitro* IC_50_) were comparable with measured titres for several HIV strains.^33^, ^34^ The suitability of predicted nAb titres as a surrogate for measured nAb titres across multiple variants, as demonstrated in this report, in turn supports the use of a predicted nAb endpoint to facilitate effective comparisons of nAb titres across variants for which the baseline nAb levels are expected to be different, because of differences in vaccine antigens and variant exposure (e.g. ancestral SARS‐CoV‐2 and contemporary Omicron subvariants).^35^, ^36^ Such cross‐variant comparisons would enable immunobridging studies to rapidly compare nAb titres associated with efficacy demonstrated against ancestral variants to neutralising titres against currently circulating variants of concern, either within a randomised clinical trial, or between new clinical trial data and historical data from past efficacy trials.

Predicted nAb titres could also facilitate definition of a nAb‐based correlate of protection for mAbs targeting SARS‐CoV‐2, which does not currently exist. Predicted nAb titres can be estimated over time using population‐PK model predictions and across variants for which nAb titres have not been measured in clinical samples, allowing for continuous assessment of titres through all SARS‐CoV‐2 variant waves. While the majority of work to date on defining correlates of protection for SARS‐CoV‐2 and other viruses has focused primarily on vaccine responses,^7^, ^8^, ^9^, ^10^, ^37^ recently published analyses of nAb titre thresholds for mAbs have indeed correlated IC_50_‐normalised serum concentrations with efficacy against symptomatic COVID‐19 for several SARS‐CoV‐2 variants.^20^, ^38^

The utilisation of serum concentrations in combination with *in vitro* IC_50_ values also allows for the prediction of nAb titres in new populations and scenarios. The effects of individual participants and disease characteristics on mAb serum concentrations and variability have been extensively characterised across different populations and indications in population‐PK models built upon pooled clinical data.^39^ As such, population‐PK model predictions and *in vitro* neutralisation data could be used together to assess predicted nAb titres for different dosing regimens in target populations and indications more quickly than clinical studies can be developed and executed.^39^, ^40^, ^41^ Such a strategy would allow for continuous review of whether current and future mAb therapeutics would be likely to maintain protection against newly emergent SARS‐CoV‐2 variants, particularly in vulnerable populations.

There are two limitations of this analysis that may limit the generalisability of the presented results. First, the current study was limited to a single mAb combination, AZD7442. Second, this dataset was collected prior to widespread availability of COVID‐19 vaccines, and therefore does not assess the impact of vaccine‐induced or naturally elicited nAb titres on the relationship between serum concentrations and nAb titres. These limitations can be addressed in future analyses of similar data from other SARS‐CoV‐2‐targeting mAbs. An additional technical limitation is the assumption of a Hill coefficient of 1 for the calculation of serum‐based IC_50_ values. This assumption was made to allow the calculation of predicted nAb based purely on serum concentrations and *in vitro* IC_50_ values, without the need for calibration on each new variant. The results here support the consistency of the serum‐based IC_50_ values with *in vitro* values, as well as predicted nAb titres with observed pseudovirus nAb titres under this set of assumptions, and confirm the utility of the presented approach to quickly estimate clinical nAb titres for new mAbs and new SARS‐CoV‐2 variants.

In conclusion, data from our analyses reported herein show that (1) pseudovirus‐based neutralisation assays are a good surrogate for authentic virus‐based assays, regardless of the SARS‐CoV‐2 viral variant; (2) variant‐specific pseudovirus nAb titres correlate strongly with AZD7442 serum concentrations across five SARS‐CoV‐2 variants; (3) serum‐based IC_50_ values are similar to *in vitro* IC_50_ measurements; and (4) predicted nAb titres derived from serum mAb concentrations in conjunction with *in vitro* IC_50_ measurements can be a surrogate for nAb titres measured in serum for each variant. Together, these data illustrate that both pseudovirus nAb titres measured in patient serum and serum mAb concentrations in combination with pseudovirus‐based *in vitro* IC_50_ values are appropriate strategies for assessing nAb titres for COVID‐19 mAbs. Therefore, these approaches offer a strategy for streamlining the clinical evaluation of mAbs targeting SARS‐CoV‐2, which has the potential to significantly decrease the time from discovery to clinical deployment of the next generation of COVID‐19 mAbs.

## Methods

### Study participants

The PROVENT study (NCT04625725) was conducted between November 2020 and November 2023. This double‐blind, placebo‐controlled phase 3 trial assessed the safety, immunogenicity and efficacy of a single dose of AZD7442 as pre‐exposure prophylaxis against COVID‐19 in individuals aged ≥ 18 years who were at increased risk of an inadequate response to COVID‐19 vaccination or had an increased risk of exposure to SARS‐CoV‐2.^17^ Participants were required to be negative for SARS‐CoV‐2 via serological testing and to be vaccine naïve. A total of 5973 participants were enrolled from 87 sites in Belgium, France, Spain, the UK and the United States, and 5197 participants were randomised in a 2:1 ratio to AZD7442 or placebo.

The TACKLE study (NCT04723394) was conducted between January 2021 and October 2022. This double‐blind, placebo‐controlled phase 3 trial assessed the safety and efficacy of AZD7442 for preventing progression to severe disease or death in non‐hospitalised participants aged ≥ 18 years with mild‐to‐moderate COVID‐19.^25^ Participants required a reverse transcription polymerase chain reaction (RT‐PCR) or antigen test‐confirmed SARS‐CoV‐2 infection and a World Health Organization Clinical Progression Scale score of > 1 to < 4, were vaccine naïve, and had to receive AZD7442 within 7 days from self‐reported onset of symptoms. The trial enrolled 1014 participants from 95 sites in the United States, Latin America, Europe and Japan, with 910 being randomised in a 1:1 ratio to AZD7442 or placebo.

The AZD7442 phase 1 first‐in‐human (NCT04507256) dose‐escalation study was conducted between August 2020 and October 2021. Full eligibility criteria have been previously described.^24^ Briefly, study participants were aged 18–55 years at the time of screening, were negative for SARS‐CoV‐2 via quantitative RT‐PCR and/or serological testing before randomisation, and were considered healthy by medical history, physical examination and baseline safety laboratory studies, according to the judgement of the investigator.

### Study approval

The PROVENT, TACKLE and AZD7442 phase 1 first‐in‐human study protocols and amendments were approved by the ethics committee or institutional review board at each participating centre. The final version of the study protocols and statistical analysis plans have been published previously and can be accessed as part of Levin *et al*. (2022),^17^ Montgomery *et al*. (2022)^25^ and Forte‐Soto *et al*. (2023),^24^ respectively. The studies were all conducted in accordance with the principles of the Declaration of Helsinki and the International Council for Harmonization Good Clinical Practice guidelines. All participants in all studies provided written informed consent before enrolment.

### Study design

The PROVENT, TACKLE and AZD7442 phase 1 first‐in‐human studies have been previously described.^17^, ^24^, ^25^ Briefly, in the PROVENT study, participants aged ≥ 18 years who were at risk of inadequate response to COVID‐19 vaccination and/or had an increased risk of exposure to SARS‐CoV‐2 were randomised 2:1 to receive AZD7442 300 mg (comprising two consecutive IM injections of tixagevimab 150 mg and cilgavimab 150 mg) or matched saline placebo. In February 2022, the US Food and Drug Administration recommended an increase in the dose of AZD7442 to 600 mg (tixagevimab 300 mg and cilgavimab 300 mg) because of the emergence of Omicron BA.1. Primary endpoints were the first episode of symptomatic COVID‐19 after exposure to AZD7442 or placebo, and safety.^17^ In the TACKLE study, unvaccinated, non‐hospitalised participants aged ≥ 18 years with mild‐to‐moderate COVID‐19 were randomised 1:1 to be dosed within 7 days of symptom onset with AZD7442 600 mg or matched saline placebo. Primary endpoints were all‐causality severe COVID‐19 or death up to Day 29, and safety throughout the course of the study.^25^ In the AZD7442 phase 1 first‐in‐human study, healthy adults aged 18–55 years were randomised 5:1 to receive a single dose of IM AZD7442 300 mg or IV AZD7442 300 mg, 1000 mg or 3000 mg. AZD7442 comprised tixagevimab and cilgavimab in a 1:1 ratio for each formulation. The primary endpoint was safety and tolerability.^24^

### Sample collection

For the analyses herein, samples were derived from the PROVENT, TACKLE and AZD7442 phase 1 first‐in‐human studies.^17^, ^24^, ^25^ In the AZD7442 phase 1 first‐in‐human study, serum samples were collected before dosing and 8 h after dosing on Day 1, and on Days 2, 4, 6, 8, 15, 31, 61, 91, 151, 211, 271 and 361. In the PROVENT study, serum samples were collected before dosing on Day 1, on Days 8, 29, 58, 92, 183 and 366, and upon discontinuation. In the TACKLE study, serum samples were collected pre‐ and post‐dose on Day 1, and on Days 3, 6, 15, 29, 85, 169 and 366. Subsets of samples based on aliquot availability were selected for the *post hoc* analysis described in this publication.

### Pharmacokinetic assessments

Bioanalytical analyses of tixagevimab and cilgavimab in serum were performed by PPD Laboratories (Richmond, VA, USA), using immunocapture to the SARS‐CoV‐2 receptor‐binding domain followed by denaturation and detection of AZD7442‐specific peptides by liquid chromatography‐mass spectrometry as described by Mu *et al*. (2022),^42^ which was validated in accordance with relevant regulatory guidelines.

### Authentic virus neutralisation assay

Serum‐neutralising titres were evaluated using the SARS‐CoV‐2 PRNT (Viroclinics Biosciences, Rotterdam, Netherlands). As described previously,^22^ a standard number of SARS‐CoV‐2 infectious units were preincubated with serial dilutions of serum for 1 h. The virus/serum mixtures were subsequently added to confluent Vero E6 cell monolayers (American Type Culture Collection, Manassas, VA, USA). After 16–24 h, cells were formalin‐fixed and then incubated with a mAb targeting the SARS‐CoV‐2 nucleocapsid protein, followed by a secondary anti‐human immunoglobulin G horseradish peroxidase conjugate (Thermo Fisher Scientific, Waltham, MA, USA) and KPL TrueBlue substrate (SeraCare Life Sciences Inc., Milford, CT, USA). The immunostained plates were scanned using an ImmunoSpot analyzer (Cellular Technology Limited, Cleveland, OH, USA), equipped with software for the quantification of nucleocapsid‐positive cells. SARS‐CoV‐2 neutralisation titres (defined by PRNT_80_, or the dilution of a sample that produced a 80% reduction in virus) were calculated from these spot counts as described by Zielinska *et al*. (2005).^43^

### Phenosense pseudovirus neutralisation assay

Neutralising titres were also assessed using the PhenoSense pseudovirus neutralisation assay (Monogram Biosciences, San Francisco, CA, USA). The PhenoSense SARS‐CoV‐2 nAb assay is based on previously described methodologies using HIV‐1 pseudovirions.^44^, ^45^ The measurement of nAb titres using the PhenoSense SARS‐CoV‐2 nAb assay was performed by generating HIV‐1 pseudovirions that express the SARS‐CoV‐2 spike protein from the virus of interest (ancestral, Alpha, Delta, Omicron BA.1, BA.2 or BA.4/5). Neutralising titres were then measured by assessing the inhibition of luciferase activity in HEK293 target cells expressing the ACE‐2 receptor, following preincubation of the pseudovirions with serial dilutions of the serum collected from trial participants or from *in vitro* dilutions of AZD7442. The expression of luciferase activity in target cells is inhibited in the presence of anti‐SARS‐CoV‐2 nAbs. Neutralising titres are reported as the reciprocal of the serum dilution conferring ID_50_ of pseudovirus infection. Assay validation for use in the clinical testing to assess neutralisation titres against ancestral SARS‐CoV‐2 and Alpha, Delta and Omicron variants included accuracy, dilutional linearity, repeatability, intermediate precision and specificity/selectivity.

### Statistical analyses

To determine the level of correlation between SARS‐CoV‐2 nAb titres derived from authentic virus and pseudovirus neutralisation assays, as well as between serum concentrations and observed nAb titres, *post hoc* correlation analyses were performed using serological data collected from the PROVENT, TACKLE and/or AZD7442 phase 1 first‐in‐human studies. To account for skewing, log_10_‐transformed values were considered. Bias‐adjusted Spearman's rank correlation estimates were used to determine the strength of the relationships, with Fisher's Z transformation used to derive 95% CIs.

A repeated measures correlation was also performed as a sensitivity analysis for authentic virus and pseudovirus neutralisation assays, as reported in Table 1, for groups where measurements from both assays were available. This was calculated following the methods by Hamlett *et al*.,^46^ and the 95% CI for the correlation coefficient was estimated using the normal approximation method by Shen and Lu.^47^ Details are given in the subsection below for the interested reader.

#### Derivation for repeated measures correlation between pseudovirus‐based and authentic virus nAb titres

For this analysis, Hamlett's linear mixed model^46^ was fitted using data from participants i from the relevant studies, at visits j using available post‐baseline measurements, with response k for either the log_10_‐transformed authentic virus nAb titres ($R_{1}$) or log_10_‐transformed pseudovirus‐based nAb titres ($R_{2}$). Covariates include an intercept and the type of response, where a value of 0 and 1 is assigned if the response variable is either $R_{1}$ or $R_{2}$, respectively. A random effect for type of response was fit at the participant level with unstructured covariance. A repeated effect was also fit for the type of response for visits nested within participant level with an unstructured covariance, to model the residual errors. The Kenward–Roger degrees of freedom method was used, and maximum likelihood estimation was performed.

The variance of the random effect has the form

$$G=\left[\begin{array}{cc} \mathrm{Co}v_{i}\left(R_{1},R_{1}\right) & \mathrm{Co}v_{i}\left(R_{1},R_{2}\right)\\ \mathrm{Co}v_{i}\left(R_{1},R_{2}\right) & \mathrm{Co}v_{i}\left(R_{2},R_{2}\right) \end{array}\right].$$

The residual variance has the form

$$R=I_{m_{i}}⊗\left[\begin{array}{cc} \mathrm{Co}v_{j\left(i\right)}\left(R_{1},R_{1}\right) & \mathrm{Co}v_{j\left(i\right)}\left(R_{1},R_{2}\right)\\ \mathrm{Co}v_{j\left(i\right)}\left(R_{1},R_{2}\right) & \mathrm{Co}v_{j\left(i\right)}\left(R_{2},R_{2}\right) \end{array}\right],$$

where $m_{i}$ is the number of observations for participant i.

The repeated measures correlation ($\hat{\rho })$ was derived as follows:

$$\hat{\rho }=\frac{\mathrm{Cov}\left(R_{1},R_{2}\right)}{\sqrt{\mathrm{Var}\left(R_{1}\right)\times \mathrm{Var}\left(R_{1}\right)}}$$

$$=\frac{\mathrm{Co}v_{i}\left(R_{1},R_{2}\right)+\mathrm{Co}v_{j\left(i\right)}\left(R_{1},R_{2}\right)}{\sqrt{\left(\left(\mathrm{Co}v_{i}\left(R_{1},R_{1}\right)+\mathrm{Co}v_{j\left(i\right)}\left(R_{1},R_{1}\right)\right)\times \left(\mathrm{Co}v_{i}\left(R_{2},R_{2}\right)+\mathrm{Co}v_{j\left(i\right)}\left(R_{2},R_{2}\right)\right)\right)}}$$

$$\frac{b+h}{\sqrt{\left(a+g\right)\times \left(c+i\right)}}$$

Applying the delta method, the partial derivative vector of $\hat{\rho }$ is defined as follows:

$$\frac{\mathrm{\partial f}}{\partial }=\left(\frac{\partial f}{\partial a}\;\frac{\partial f}{\partial b}\;\frac{\partial f}{\partial c}\;\begin{array}{ccc} \frac{\partial f}{\partial g} & \frac{\partial f}{\partial h} & \frac{\partial f}{\partial i} \end{array}\right),$$

where

$$\frac{\partial f}{\partial a}=\frac{\partial f}{\partial g}=-\frac{1}{2}\frac{\left(b+h\right)\left(c+i\right)}{\sqrt{{\left[\left(a+g\right)\times \left(c+i\right)\right]}^{3}}}$$

$$\frac{\partial f}{\partial b}=\frac{\partial f}{\partial h}=\frac{1}{\sqrt{\left(a+g\right)\times \left(c+i\right)}}$$

$$\frac{\partial f}{\partial c}=\frac{\partial f}{\partial i}=-\frac{1}{2}\frac{\left(b+h\right)\left(a+g\right)}{\sqrt{{\left[\left(a+g\right)\times \left(c+i\right)\right]}^{3}}}$$

The 95% CI was derived as follows:

$$\left(\hat{\rho }-1.96\times \sqrt{\left(\frac{\mathrm{\partial f}}{\partial }\Sigma {\frac{\mathrm{\partial f}}{\partial }}^{T}\right)},\hat{\rho }+1.96\times \sqrt{\left(\frac{\mathrm{\partial f}}{\partial }\Sigma {\frac{\mathrm{\partial f}}{\partial }}^{T}\right)}\right),$$

where Σ is the asymptotic covariance from the linear mixed model.

#### Calculation of fold difference for pseudovirus and authentic virus nAb assays

In Figure 2, estimated fold difference values were calculated from nAb titres for pseudovirus and authentic virus assays, and simple linear regression lines are presented for visualisation. A normally distributed generalised linear mixed effect model was fitted for each participant i and timepoint j, using the form

$$Y_{i,j}|U_{i}\sim N\left(\mu_{i,j},\sigma^{2}\right),U_{i}\sim N\left(0,G\right),$$

where $Y_{i,j}=\log_{10}\left(\text{pseudovirus}\;\mathrm{nAb}\;\text{titr}e_{i,j}\right)$; $\mu_{i,j}=\beta_{0}+\log_{10}\left(\text{authentic virus neutralising titr}e_{i,j}\right)$; G random participant unstructured covariance matrix.

The 1:1 relationship (linear slope = 1) was assumed to evaluate the overall fold difference between the two methods. From this model, the fold difference was derived as $10^{\beta_{0}}$, with the same transformation applied to the 95% confidence limits of $\beta_{0}$ to derive the 95% CI.

#### Calculation of serum‐based IC_50_ values

To estimate serum‐based AZD7442 IC_50_ values from serum mAb concentrations and observed nAb titre data for variant k, the assumed relationship to fit is

$$\mathrm{nAb}\;\text{titr}e_{k}=\frac{\text{Serum concentration}\;\left(\mathrm{ng}\;{\mathrm{mL}}^{–1}\right)\;}{I{C_{50}}_{k}\;\left(\mathrm{ng}\;{\mathrm{mL}}^{–1}\right)}.$$

This model form assumes a Hill coefficient of 1 for each variant, in order to allow the calculation of predicted nAb titres based on serum concentrations and *in vitro* IC_50_ values alone. Using a more complex relationship between serum concentration and pseudovirus nAb (including the exponential term that would correspond to the slope on the log–log scale) would necessitate the collection of observed nAb data for each variant to fit the relationship in the future.

We expect residuals for nAb titres and serum concentration to be log‐normally distributed. Therefore, to model this we consider the form:

$$\log_{10}\left(\mathrm{nAb}\;\mathrm{tit}{\mathrm{re}}_{k}\right)=\log_{10}\left(\text{Serum concentration}\;(\left[\mathrm{ng}\;{\mathrm{mL}}^{–1}\right]\right)-\log_{10}\left(I{C_{50}}_{k}\right)).$$

A normally distributed generalised linear mixed effect model was fitted for each participant i, timepoint j, and variant k using the form:

$$Y_{i,j,k}|U_{i}\sim N\left(\mu_{i,j,k},\sigma^{2}\right),U_{i}\sim N\left(0,G\right),$$

where $Y_{i,j,k}=\log_{10}\left(\mathrm{nAb}\;\text{titr}e_{i,j,k}\right)$; $\mu_{i,j,k}=\beta_{k}+\log_{10}(\text{Serum}\text{concentration}\;({\left[\mathrm{ng}\;{\mathrm{mL}}^{–1}\right]}_{i,j}))$; G random participant unstructured covariance matrix.

From this model, $\beta_{k}=-\log_{10}\left(I{C_{50}}_{k}\right)$; therefore, $I{C_{50}}_{k}=10^{\left(-\beta_{k}\right)}$. Given this is a monotonic transformation, the 95% CI can be derived from back transformation. The same back transformation was applied to the confidence limits of $\beta_{k}$ to derive the 95% CI.

In Figure 3a and Supplementary figures 1 and 2a, the predicted nAb titre line is plotted using serum AZD7442 concentrations and predicted nAb titres. In Figure 3b and Supplementary figure 2b, the line *y* = *x* is plotted to compare agreement between the observed and predicted nAb titres. All PK and nAb titre measurements below the LLoQ were imputed to half of the LLoQ for visualisation but were excluded in correlation analyses, unless specified.

## Author contributions

**Lindsay E Clegg:** Conceptualization; data curation; formal analysis; investigation; methodology; project administration; data visualization; writing – original draft; writing – review and editing. **Oleg Stepanov:** Data curation; formal analysis; methodology; data visualization; writing – review and editing. **Sam Matthews:** Formal analysis; methodology; software; data validation; writing – review and editing. **Tom White:** Formal analysis; methodology; software; data validation; data visualization; writing – review and editing. **Seth Seegobin:** Formal analysis; data validation; writing – review and editing. **Steven Thomas:** Formal analysis; data validation; writing – review and editing. **Kevin M Tuffy:** Conceptualization; formal analysis; data visualization; writing – review and editing. **Mats Någård:** Conceptualization; investigation; supervision; data validation; writing – review and editing. **Mark T Esser:** Conceptualization; investigation; methodology; supervision; writing – review and editing. **Katie Streicher:** Conceptualization; methodology; data validation; writing – review and editing. **Taylor S Cohen:** Conceptualization; investigation; supervision; writing – original draft; writing – review and editing. **Anastasia A Aksyuk:** Conceptualization; data curation; formal analysis; investigation; methodology; project administration; supervision; data visualization; writing – original draft; writing – review and editing.

## Conflict of interest

The authors of this report are all current employees of AstraZeneca and may own AstraZeneca stock or stock options. SM is a contractor to AstraZeneca via Exploristics (Belfast, UK). MTE is a named inventor on patents planned, issued or pending relating to AZD7442.

## Supporting information


Supplementary figure 1
Supplementary figure 2
Supplementary table 1

## Data Availability

Data underlying the findings described in this manuscript may be obtained in accordance with AstraZeneca's data sharing policy described at https://www.astrazenecaclinicaltrials.com/our‐transparency‐commitments/. Data for studies directly listed on Vivli can be requested through Vivli at www.vivli.org. Data for studies not listed on Vivli can be requested through Vivli at https://vivli.org/members/enquiries‐about‐studies‐not‐listed‐on‐the‐vivli‐platform/. The AstraZeneca Vivli member page is also available outlining further details: https://vivli.org/ourmember/astrazeneca/.
