# Supplementary material for: Serum AZD7442 (tixagevimab–cilgavimab) concentrations and in vitro IC50 values predict SARS‐CoV‐2 neutralising antibody titres
Source: Clin Transl Immunology. 2024 Jun 13;13(6):e1517. doi: 10.1002/cti2.1517 (PMC11175839; doi:10.1002/cti2.1517)
Supplement: Supplementary file 1 — Supplementary figure 1 Supplementary figure 2 Supplementary table 1 [file CTI2-13-e1517-s001.docx]

**SUPPORTING INFORMATION**

**
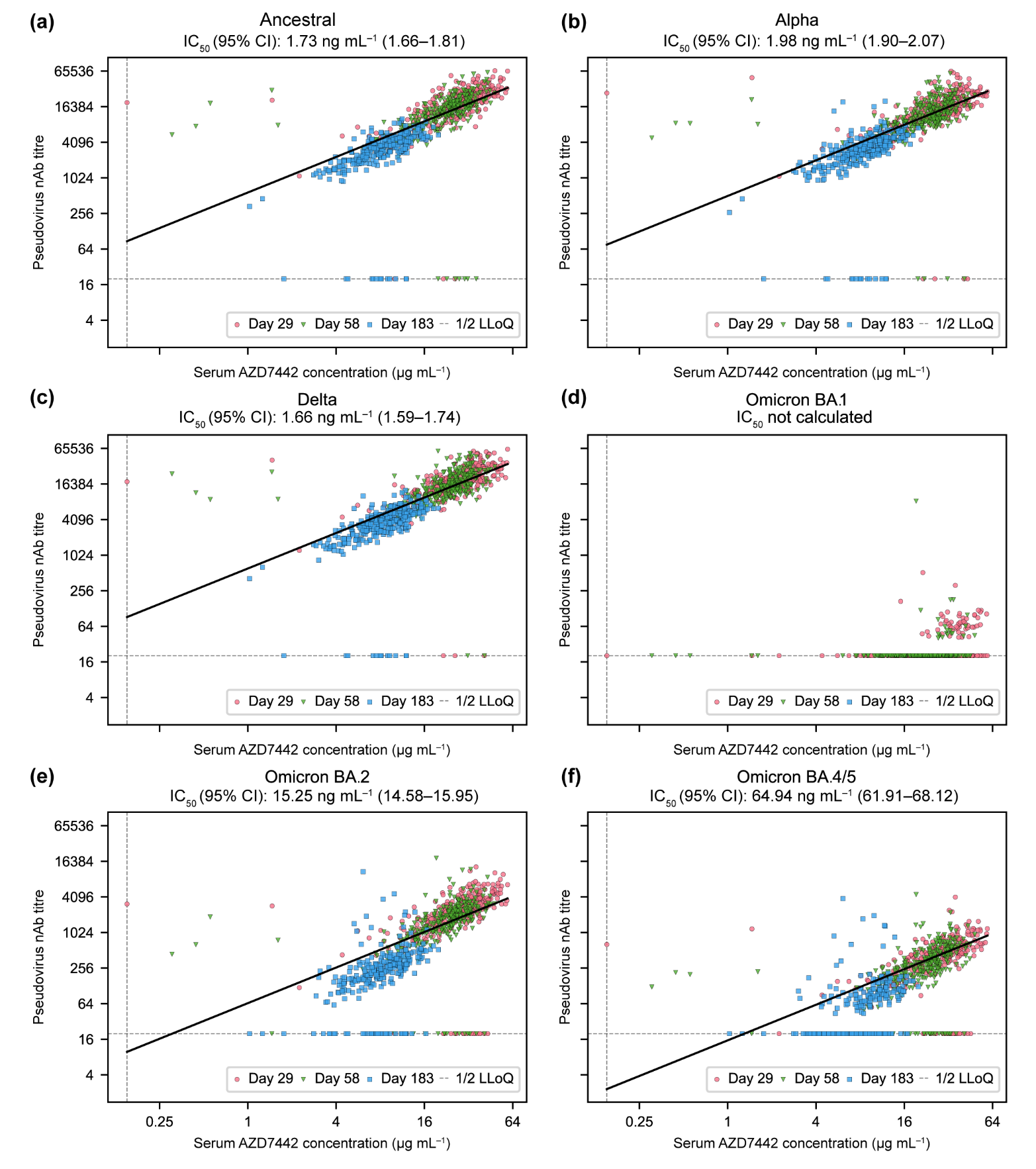
**

**Supplementary figure 1.** Correlation of AZD7442 PK and nAb titres from a subset of participants in PROVENT by SARS-CoV-2 variant. *Post-hoc* correlation analysis depicting the relationship between nAb titres determined by pseudovirus assay (*y*-axis) for ancestral SARS-CoV-2, and SARS-CoV-2 Alpha, Delta, Omicron BA.1, Omicron BA.2 and Omicron BA.4/5, and serum concentrations of AZD7442 (*x*-axis). Datapoints depict matched PK and nAb sample pairs (observations) from the same PROVENT study participants. The black solid line on each graph is the fitted predicted nAb titre line derived from the serum based IC_50_ for each variant (values below the LLoQ were excluded). The Spearman correlations for each variant with 95% CIs were calculated using the Fisher transformation and are shown above each graph. PK and nAb titre measurements below the LLoQ were imputed to half of the LLoQ for visualisation.

CI, confidence interval; IC_50_, half maximal inhibitory concentration; LLoQ, lower limit of quantification; nAb, neutralising antibody; PK, pharmacokinetics; SARS-CoV-2, severe acute respiratory coronavirus 2.


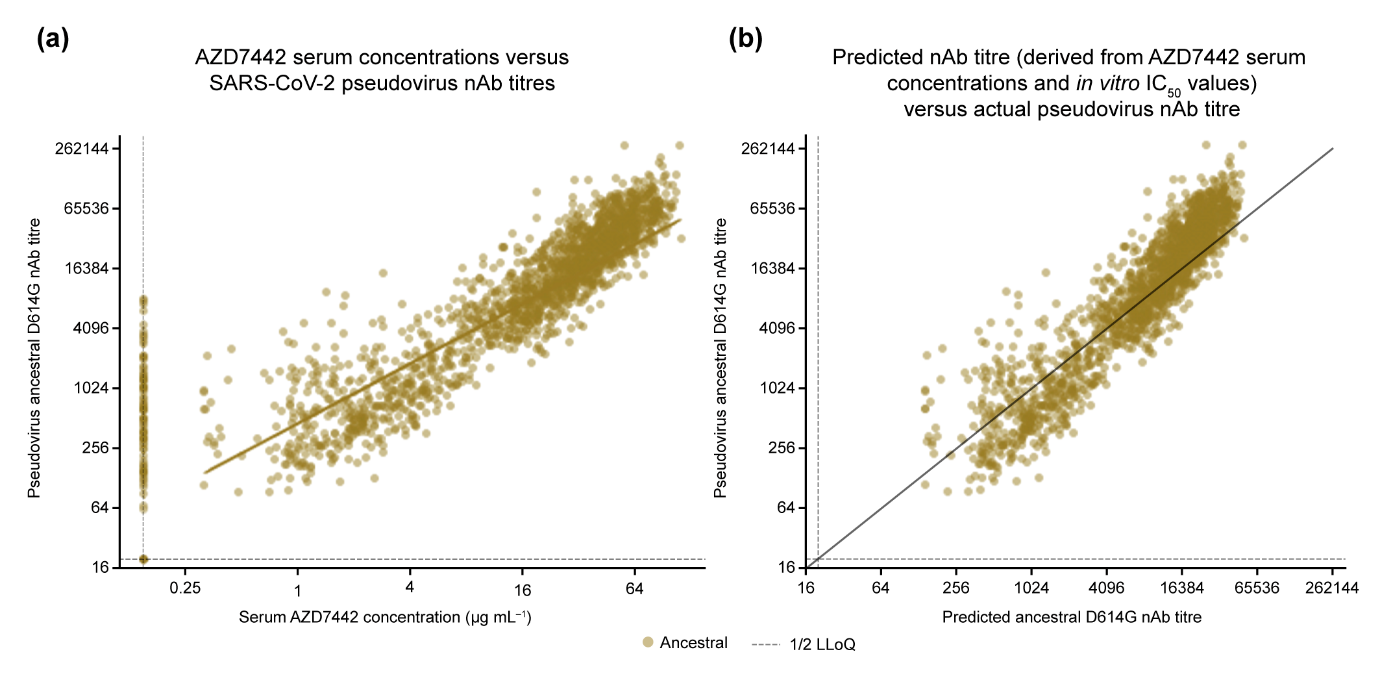


**Supplementary figure 2.** Correlation of serum AZD7442 concentrations and SARS-CoV-2 pseudovirus nAb titres against the ancestral SARS-CoV-2 virus in TACKLE study participants. *Post-hoc* correlation analysis depicting the relationship between nAb titres determined by **(a)** pseudovirus assay and serum concentrations of AZD7442 or **(b)** predicted nAb titres. Data points depict matched PK and nAb sample pairs (observations) from the same study participants. PK and nAb titre measurements below the LLoQ were imputed to half of the LLoQ for visualisation in **a.**

LLoQ, lower limit of quantification; nAb, neutralising antibody; PK, pharmacokinetic.

**Supplementary table 1*.*** Spearman correlations for serum AZD7442 concentrations and pseudovirus nAb titres across SARS-CoV-2 variants in PROVENT study participants when measurements below the LLoQ are included and imputed to 1/2 LLoQ

| SARS-CoV-2 virus | n^†^ | Spearman correlation (95% CI)^‡^ |
| --- | --- | --- |
| Ancestral | 879 | 0.86 (0.84–0.88) |
| Alpha | 897 | 0.84 (0.82–0.86) |
| Delta | 897 | 0.85 (0.83–0.87) |
| Omicron BA.1^§^ | 595 | NE |
| Omicron BA.2 | 880 | 0.77 (0.74–0.80) |
| Omicron BA.4/5 | 880 | 0.71 (0.67–0.74) |

^†^ Number of available post-baseline observations for both pseudovirus nAb titres and serum concentration for calculation of Spearman correlations.
^‡^ As log_10_-transformed nAbs and serum concentrations are skewed, the bias-adjusted Spearman correlation was used following Fisher’s Z transformation for the 95% CI.
^§^ As a high percentage of observations were below the LLoQ for Omicron BA.1, it was deemed there was not sufficient data to evaluate the serum concentration and nAb correlation.

CI, confidence interval; nAb, neutralising antibody; LLoQ, lower limit of quantification; NE, not evaluable; SARS-CoV-2, severe acute respiratory coronavirus 2.
